# The Effects of Structural Alterations in the Polyamine and Amino Acid Moieties of Philanthotoxins on Nicotinic Acetylcholine Receptor Inhibition in the Locust, *Schistocerca gregaria*

**DOI:** 10.3390/molecules26227007

**Published:** 2021-11-19

**Authors:** Victoria L. Luck, David P. Richards, Ashif Y. Shaikh, Henrik Franzyk, Ian R. Mellor

**Affiliations:** 1 School of Life Sciences, University of Nottingham, University Park, Nottingham NG7 2RD, UK; toriluck94@gmail.com (V.L.L.); Richards.d.p@outlook.com (D.P.R.); 2 Department of Drug Design and Pharmacology, University of Copenhagen, Jagtvej 162, 2100 Copenhagen, Denmark; ashif@sund.ku.dk (A.Y.S.); henrik.franzyk@sund.ku.dk (H.F.)

**Keywords:** philanthotoxin, nicotinic acetylcholine receptor, locust, neuron, patch-clamp

## Abstract

Alterations in the polyamine and amino acid (tyrosine) moieties of philanthotoxin-343 (PhTX-343) were investigated for their effects on the antagonism of nicotinic acetylcholine receptors (nAChRs) isolated from the locust (*Schistocerca gregaria*) mushroom body. Through whole-cell patch-clamp recordings, the philanthotoxin analogues in this study were shown to cause inhibition of the inward current when co-applied with acetylcholine (ACh). PhTX-343 (IC_50_ = 0.80 μM at −75 mV) antagonised locust nAChRs in a use-dependent manner, suggesting that it acts as an open-channel blocker. The analogue in which both the secondary amine functionalities were replaced with methylene groups (i.e., PhTX-12) was ~6-fold more potent (IC_50_ (half-maximal inhibitory concentration) = 0.13 μM at −75 mV) than PhTX-343. The analogue containing cyclohexylalanine as a substitute for the tyrosine moiety of PhTX-343 (i.e., Cha-PhTX-343) was also more potent (IC_50_ = 0.44 μM at −75 mV). A combination of both alterations to PhTX-343 generated the most potent analogue, i.e., Cha-PhTX-12 (IC_50_ = 1.71 nM at −75 mV). Modulation by PhTX-343 and Cha-PhTX-343 fell into two distinct groups, indicating the presence of two pharmacologically distinct nAChR groups in the locust mushroom body. In the first group, all concentrations of PhTX-343 and Cha-PhTX-343 inhibited responses to ACh. In the second group, application of PhTX-343 or Cha-PhTX-343 at concentrations ≤100 nM caused potentiation, while concentrations ≥ 1 μM inhibited responses to ACh. Cha-PhTX-12 may have potential to be developed into insecticidal compounds with a novel mode of action.

## 1. Introduction

**Figure 1:**
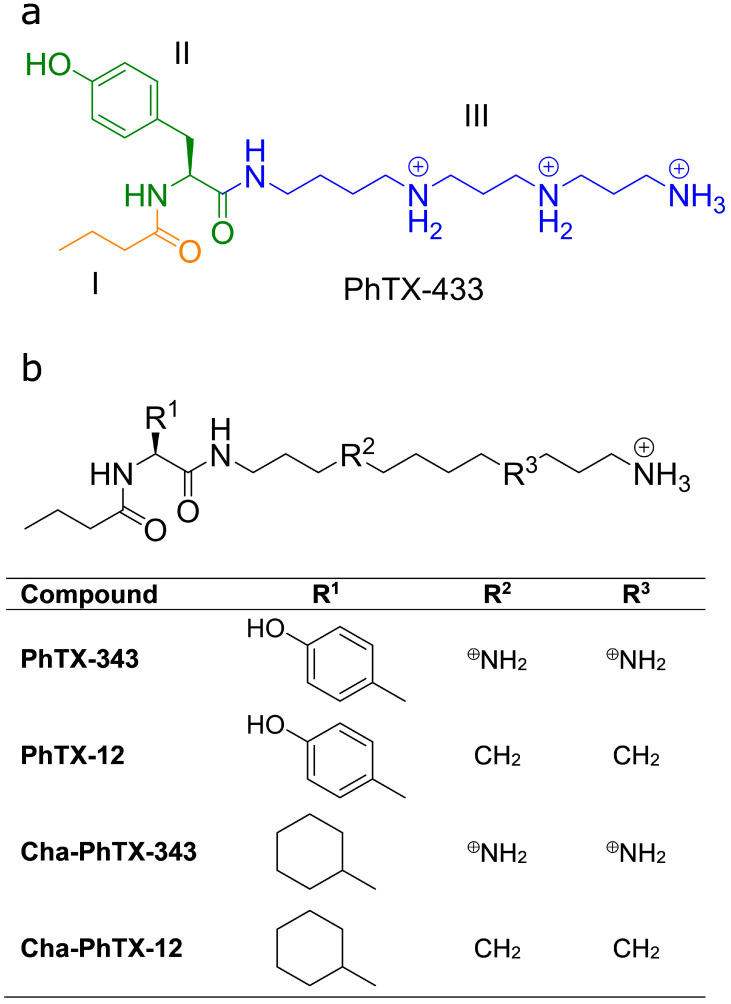
Structure of PhTX-433 and analogues: (**a**) Structure of the naturally occurring PhTX-433 from the venom of *Philanthus triangulum*. The modules of the structure comprise a butyryl group (I, orange), a central tyrosyl residue (II, green), and a polyamine (thermospermine) moiety (III, blue). (**b**) The compounds assessed in this study.

Venoms from many predatory wasps and spiders contain polyamine toxins that have been used as pharmacological tools in the study of ionotropic receptors, but their value has been limited due to a lack of selectivity [1]. Among these polyamine toxins, philanthotoxins (PhTXs) are of particular interest. Philanthotoxin-433 (PhTX-433; Figure 1a) is a naturally occurring polyamine-containing toxin found as a constituent in the venom of the Egyptian digger wasp, *Philanthus triangulum*. The modular structure of PhTX-433 (Figure 1a) facilitates the development of novel synthetic analogues that can be made specific against particular ionotropic receptors [2]. PhTX-433 and its synthetic analogues are non-competitive antagonists of ionotropic glutamate receptors of vertebrate nervous systems and insect muscles, as well as of nicotinic acetylcholine receptors (nAChR), expressed by the cells of a variety of organisms, including human muscles [3,4], the mammalian central nervous system (CNS) [5,6,7], and the insect CNS [8].

The nAChR is a non-selective cation channel found throughout the central and peripheral nervous systems of vertebrates and invertebrates, the skeletal muscles in vertebrates [3], as well as in some cells of the immune system and cancer cells. In insects they are expressed solely in the nervous system and are especially abundant in excitatory post-synaptic membranes [9], where they play a major role as mediators of fast excitatory synaptic transmission [10]. The functional architecture and structure of insect nAChRs are less well understood, and models of the insect nAChR are based on knowledge of their vertebrate counterparts.

Although they share many physiological and pharmacological properties with vertebrate receptors [11], insect nAChRs display increased sensitivity to certain compounds, and as such present an important insecticide target [12]. Pesticides that target cholinergic neurotransmission are highly effective. They account for a significant share of the world insecticide market and include one of the most widely used classes of insecticides, the neonicotinoids. Unfortunately, pervasive use of these few insecticide classes has placed a strong selection pressure on target organisms [13], which inevitably has resulted in the development of resistance in pest populations [14]. However, insect nAChRs remain a highly attractive target for insecticide discovery [10,15,16,17,18], and the relative ease of synthesising new PhTX analogues [19] creates potential for the development of insect nAChR-selective antagonists [20].

Structure–activity studies of PhTX-343, a synthetic model compound for PhTX-433, have shown that each moiety plays a role in determining the potency of PhTXs towards ionotropic receptors [4,5,21,22,23,24,25,26,27]. In particular, a certain degree of hydrophobicity of the headgroup (Figure 1a, Regions I and II) is important for high potency toward nAChRs [5,22], while removal of the secondary amines from the polyamine moiety (Figure 1a, Region III) of PhTX-343 (to give PhTX-12) increases potency toward human muscle-type nAChRs [3,27], albeit this alteration greatly reduces potency toward some insect nAChRs [22,28]. For vertebrate nAChRs, voltage-dependence studies have revealed that, in addition to different potencies, PhTX-343 and PhTX-12 have different binding sites and modes of action. PhTX-343 predominantly acts as a voltage-dependent open-channel blocker that penetrates deep into the pore [29], while PhTX-12 predominantly inhibits in a voltage-independent way by interacting with the hydrophobic upper region of the pore when the nAChR is in a closed state [3].

The present study investigates how removal of the secondary amine functionalities from the polyamine tail of PhTX-343 and replacement of the tyrosyl moiety with cyclohexylalanine (Figure 1b) influences the potency of the resulting PhTX analogues toward nAChRs isolated from the locust, *Shistocerca gregaria,* brain mushroom bodies. Due to the highly conserved architecture of nAChRs among species [30], insect nAChRs were expected to respond to PhTX analogues in a similar way to vertebrate nAChRs. Here, we show that double-modified Cha-PhTX-12 is a highly potent antagonist of locust brain nAChRs, and as such it may facilitate design of a PhTX analogue that is selective for insect nAChRs, thus having potential for being developed into an insecticide.

## 2. Results

### 2.1. Synthesis of Cha-PhTX-12

Starting from mono-Boc protected 1,12-diaminododecane [27], successive acylation with the activated pentafluorophenyl ester Fmoc-Cha-OPfp [21], Fmoc deprotection with octanethiol and 1,8-diazabicyclo[5.4.0]undec-7-ene (DBU), and a final acylation with C_3_H_7_COOPfp [27] (see Scheme S1 in Supplementary Material) afforded Cha-PhTX-12.

### 2.2. Characterisation of Whole-Cell Current Responses to ACh in Locust Neurons

**Figure 2:**
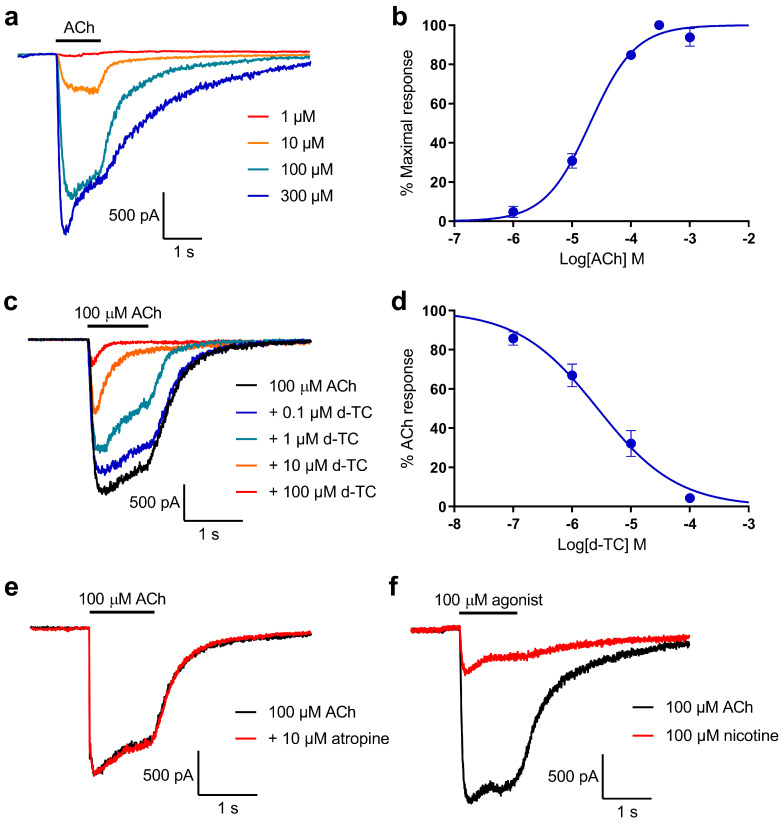
Characterisation of ACh-evoked whole-cell currents in locust neurons: (**a**) Whole-cell current recordings from a neuron exposed to ACh at 1, 10, 100 and 300 μM; (**b**) Concentration–response curve for ACh. The points are mean peak response to ACh ± SEM (*n* = 11), and the resulting curve is a fit of Equation (1); (**c**) Sample whole-cell currents in response to 100 μM ACh in the absence and presence of *d*-tubocurarine (*d*-TC) at 0.1 to 100 μM; (**d**) Concentration–response curve for inhibition of 100 μM ACh responses by *d*-tubocurarine. The points are mean % of control response to ACh ± SEM (*n* = 11), and the curve is a fit of Equation (2); (**e**) Whole-cell current responses to 100 μM ACh in the absence (black) and presence (red) of 10 μM atropine; (**f**) Whole-cell current responses to 100 μM ACh (black) and 100 μM nicotine from the same neuron.

Application of ACh to locust neurons produced robust whole-cell current responses at concentrations above 1 μM (Figure 2a). The responses were stable at lower concentrations, while at higher concentrations they demonstrated a peak followed by a relatively slow decay to a substantial plateau due to moderate desensitisation. The ACh concentration–response relationship (Figure 2b) allowed estimation of its EC_50_ (half-maximal effective concentration) as 20.3 μM (95% CI (confidence interval), 16.2–25.3 μM; *n* = 11). Further experiments with the antagonism of ACh responses utilised 100 μM, providing a strong but submaximal whole-cell current.

The co-application of 0.1 to 100 μM *d*-tubocurarine with 100 μM ACh caused inhibition of the current (Figure 2c); the concentration–inhibition curve (Figure 2d) provided an estimate of the IC_50_ (half-maximal inhibitory concentration) of 2.73 μM (95% CI, 1.75–4.27 μM; *n* = 11). At the highest concentration of *d*-tubocurarine, complete inhibition could be obtained. In contrast, co-application of 10 μM atropine (studies on insect muscarinic acetylcholine receptors show K_i_ values in the low nM range) with 100 μM ACh had no effect on the ACh response (Figure 2e). Neurons that responded to 100 μM ACh also responded to 100 μM nicotine, although the response was smaller than that seen for ACh (Figure 2f). Combined, these data show that the whole-cell currents of locust neurons in response to ACh are entirely mediated by nAChRs.

### 2.3. The Impact of Modification to the Polyamine Region

**Figure 3:**
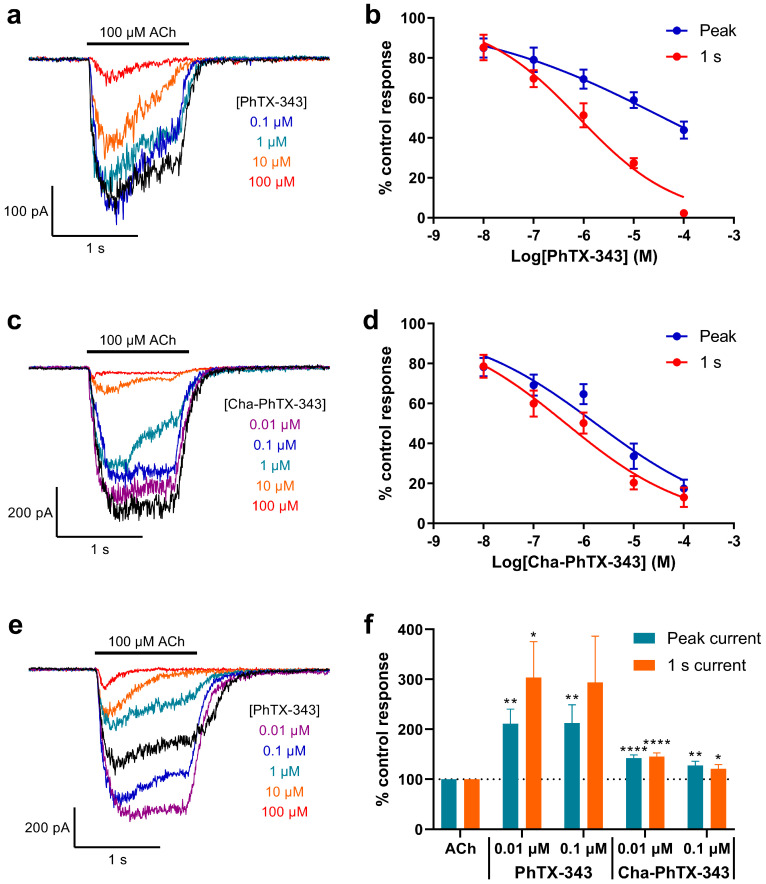
Inhibition and potentiation of locust neuronal nAChR by tribasic PhTX analogues: (**a**,**c**,**e**) Sample whole-cell currents in response to 100 μM ACh in the absence (black) and presence of PhTX-343 (**a**,**e**) or Cha-PhTX-343 (**c**) at 0.1 to 100 μM (and 0.01 μM in e). V_H_ was −75 mV; (**b**,**d**) Concentration–inhibition relationships for inhibition of 100 μM ACh evoked currents at their peak (blue), or after 1 s of activation (red) by PhTX-343 (*n* = 15) (**b**) or Cha-PhTX-343 (*n* = 10) (**d**). Points are mean % of the control ACh response ± SEM. The curves were obtained by fitting Equation 2. IC_50_ values are given in Table 1; (**f**) Potentiation of peak (purple) and 1 s (green) 100 μM ACh-evoked current by low concentrations of PhTX-343 (*n* = 8) and Cha-PhTX-343 (*n* = 12). Bars are mean % of control ACh response ± SEM. * *p* < 0.05, ** *p* < 0.01, **** *p* < 0.0001.

**Figure 4:**
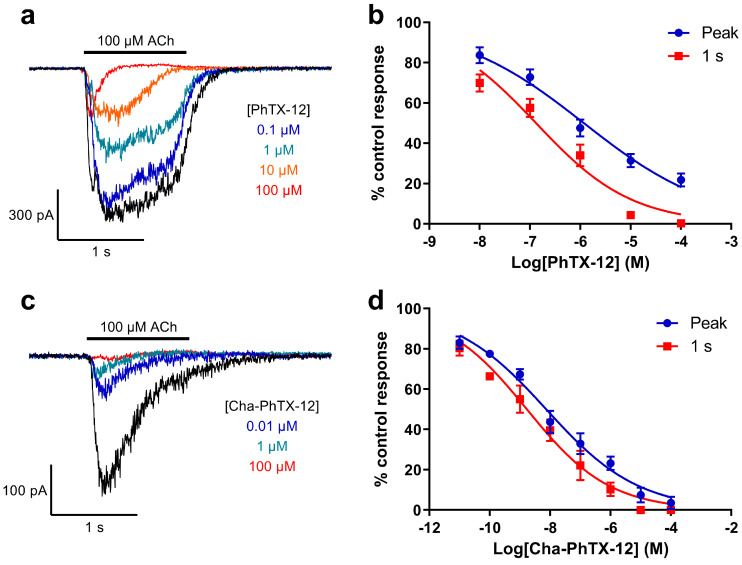
Inhibition of locust neuronal nAChR by monobasic analogues: (**a**,**c**) Sample whole-cell currents in response to 100 μM ACh in the absence (black) and presence of PhTX-12 at 0.1 to 100 μM (**a**), or Cha-PhTX-12 at 0.01 to 100 μM (**c**). V_H_ was −75 mV. (**b**,**d**) Concentration–inhibition relationships for inhibition of 100 μM ACh-evoked currents at their peak (blue), or after 1 s of activation (red) by PhTX-12 (*n* = 19) (**b**) or Cha-PhTX-12 (*n* = 7) (**d**). Points are mean % of the control ACh response ± SEM. The curves are fits of Equation (2). IC_50_ values are given in Table 1.

PhTX-343 inhibited ACh-evoked whole-cell currents with a strong dependence on activation of the nAChRs, since the IC_50_ determined after 1 s of nAChR activation was about 50-fold (IC_50_, 39.8/0.8) lower than that determined for the peak current (Figure 3a,b; Table 1). Replacement of the two secondary amine functionalities in the polyamine chain (generating PhTX-12) caused a 6.2-fold (IC_50_, 0.80/0.13) increase in potency (*p* < 0.0001) for inhibition of the 1 s current, but a 34-fold (IC_50_, 39.8/1.17) increase (*p* < 0.0001) for inhibition of the peak current, meaning that use/activation dependence (peak current inhibition/1 s current inhibition) was less pronounced (9-fold; IC_50_, 1.17/0.13) for PhTX-12 (Figure 4a,b; Table 1).

### 2.4. The Effect of Replacing the Tyrosyl Moiety

Exchanging the tyrosyl moiety of PhTX-343 for cyclohexylalanyl, producing Cha-PhTX-343, conferred a mildly increased potency in the inhibition of the 1 s current by 1.8-fold (IC_50_, 0.80/0.44; *p* = 0.184), whilst, as for PhTX-12, the potency increase for peak current inhibition was greater (23-fold (IC_50_, 39.8/1.75); *p* < 0.0001) (Figure 3c,d; Table 1); this implies a lower dependence on the use/activation of the nAChRs (4-fold; IC_50_, 1.75/0.44).

### 2.5. The Effect of Combined Modification in the Polyamine and Amino Acid Moieties

When the removal of secondary amine groups from the polyamine was combined with an exchange of the tyrosyl group for cyclohexylalanyl, producing Cha-PhTX-12, a substantial increase in potency was seen. For inhibition of the 1 s current the IC_50_ was 471-fold (IC_50_, 0.80/0.0017) lower than that for PhTX-343 and it was >5000-fold (IC_50_, 39.8/0.0075) lower for inhibition of the peak current (Figure 4c,d; Table 1). The use/activation dependence was reduced 4.4-fold (IC_50_, 0.0075/0.0017).

### 2.6. Low Concentrations of PhTX-343 and Cha-PhTX-343 Potentiate Responses to ACh in Some Cells

In some cells, co-application of 0.01 and 0.1 μM PhTX-343 and Cha-PhTX-343 caused a significant potentiation of the whole-cell current in response to ACh (Figure 3e,f). This was observed in 8 out of 23 cells for PhTX-343, and in 12 out of 22 cells for Cha-PhTX-343. In the same cells, concentrations of 1 μM and higher of these toxins caused inhibition that was similar to that in the remaining cells. For PhTX-343, at both 0.01 and 0.1 μM a mean potentiation of around 200% and 300% of the ACh control response was seen for the peak and 1 s current, respectively (Figure 3f). For Cha-PhTX-343, potentiation was similar for the peak and 1 s current, but it was lower than that for PhTX-343 at about 145% and 125% at 0.01 and 0.1 μM, respectively (Figure 3f).

## 3. Discussion

We demonstrated that all of the PhTX analogues tested here are inhibitors of the nAChRs expressed in the neurons of locust brain mushroom bodies. For the reference compound, PhTX-343, this was not surprising as it was previously found to inhibit insect nAChRs from locusts and cockroaches [22]. Additionally, it was expected that PhTX-12 might be more potent than PhTX-343, as this was demonstrated previously in mammalian nAChRs [3,4,6,27,31]. In contrast, it was not envisaged that Cha-PhTX-343 would be only slightly (and not statistically significantly) more potent than PhTX-343 for the 1 s current; moreover, Cha-PhTX-343 proved less potent (*p* = 0.0075) than PhTX-12. Previously, Cha-PhTX-343 was reported to be a particularly strong antagonist of nAChRs, with IC_50_ values in the low nanomolar range against rat subtypes α4β2 and α3β4, nAChRs [5], and >100 and >10 times more potent than PhTX-343 and PhTX-12, respectively, against human muscle-type nAChRs [25]. Interestingly, the potency of Cha-PhTX-12 has not previously been assessed with any nAChR subtype and, as expected, combination of both the above structural modifications proved to have a synergistic effect on its potency, resulting in a 471-fold (for 1 s current) increased potency compared to that of PhTX-343.

**Figure 5:**
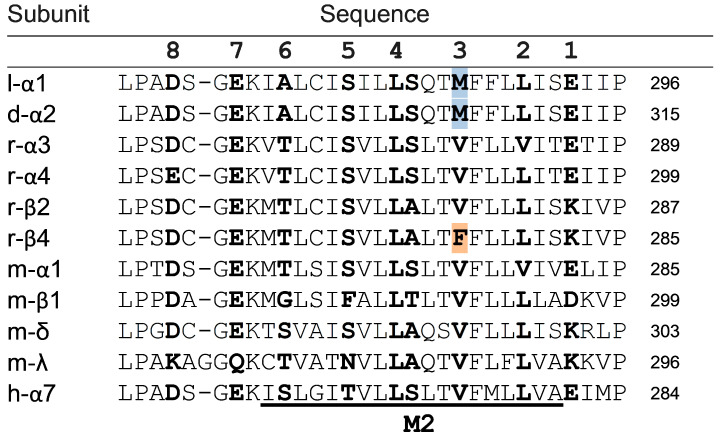
Alignment of nAChR amino acid sequences for the M1–M2 loop and M2 region of the *S. gregaria α*1 subunit (l-α1), *Drosophila melanogaster α*2 subunit (d-α2), and various vertebrate (r = rat, m = mouse, h = human) α and non-α subunits. Residues expected to line the nAChR pore (termed rings below) are depicted in bold and numbered 1–8, starting at the extracellular terminus. In ring 3, M of l-α1 and d-α2 are highlighted blue, while F of r-β4 (a difference that is conserved in other vertebrate species including humans) is highlighted in orange. The numbers at the end of the row are the residue numbers of the conserved proline at the end of each sequence.

A model for the binding of PhTX analogues to nAChRs [29] proposes that compounds displaying three amine groups in the polyamine moiety (i.e., tribasic PhTXs) remain extended during binding, and that these charged groups interact through electrostatic interactions with amino acids (e.g., S and T; Figure 5, rings 5 and 6) deep within the pore region, following the opening of the pore. Meanwhile, the bulkier hydrophobic headgroup (i.e., moieties I and II) interacts with the wider outer parts of the pore which is lined by rings of hydrophobic amino acids (Figure 5, rings 2 and 3). Compounds with a single terminal amine tend to adopt a folded structure, and they are thought to interact predominantly with the hydrophobic outer part of the pore, with binding taking place prior to opening of the pore [29] as it is above the equatorial leucine gate (Figure 5, ring 4). This mode of binding appears to be preferred for more hydrophobic analogues of PhTX-343; hence, these analogues demonstrate less use dependence, which also is supported by our data. Cha-PhTX-12, being strongly hydrophobic, clearly fits particularly well within this binding site, where it can form extensive hydrophobic interactions with rings 2 and 3 (Figure 5). These interactions may be reinforced by the unique presence of a methionine residue (M286) in a central location of the outer pore in the *S. gregaria* nAChR αL1 subunit (and in *Drosophila melanogaster* α2, *Myzus persicae* α1, and other insect α-subunits) (Figure 5). This site is normally occupied by valine, except in vertebrate β4 subunits, where a phenylalanine residue generates selectivity for PhTX analogues containing aromatic rings in their headgroup (moieties I and II) due to π-π interactions [5,6].

The strong potentiation of responses to ACh in some cells by low concentrations of the two compounds containing spermine as moiety III has not been reported previously. However, it is known that spermine alone is capable of potentiating responses of human muscle-type nAChRs to ACh at lower concentrations before inhibition at higher spermine concentrations [32]. This suggests that some types of neurons from the locust mushroom bodies express a nAChR with unique pharmacological properties, including the ability to be potentiated by spermine-containing compounds. The physiological impact of this potentiation may cause an intoxicated insect to experience rigid paralysis as opposed to the flaccid paralysis expected for nAChR antagonists. This may be a further exploitable property of these molecules.

In conclusion, combining two potency-enhancing modifications of PhTX-343 resulted in a compound, Cha-PhTX-12, which is highly tuned for the inhibition of *S. gregaria* nAChRs when compared to PhTX-12 and Cha-PhTX-343 displaying single modifications. Therefore, Cha-PhTX-12 may constitute a lead for insecticidal compounds with a unique mode of action that is able to paralyse pest insects, pending our further work on non-insect nAChRs. The strong potentiation by spermine-containing compounds may also be an exploitable feature in insecticide design.

## 4. Materials and Methods

### 4.1. Philanthotoxin Analogues

The syntheses of PhTX-343, PhTX-12, and Cha-PhTX-343 have been described previously by Wellendorph et al. [33] and Olsen et al. [25]. The novel analogue Cha-PhTX-12 was prepared via a solution-phase protocol [27] as described in the Supplementary Material.

### 4.2. Locust Neuron Preparation and Cell Culture

Neuronal cells were harvested through dissection of adult desert locusts (*Shistocerca gregaria*) purchased from livefoods.com. Following 10 min of cold anaesthesia at 4 °C, the locusts were submerged in 70% ethanol and their heads removed by decapitation with dissection scissors. The front of the head capsule was removed to expose the mushroom body of the brain, which was extracted, cut into small pieces, and placed into a 1 mL Eppendorf tube with 200 μL of Rinaldi’s saline (135 mM NaCl, 25 mM KCl, 0.4 mM NaHCO_3_, 0.5 mM glucose, and 5 mM HEPES adjusted to pH 7.2 with NaOH) containing 0.5 mg/mL dispase and 2 mg/mL collagenase. After 15 min incubation at 36.5 °C, neurons were centrifuged at 800× *g* for 1 min at room temperature to form a pellet. The supernatant was removed and replaced with 200 μL locust culture medium (5:4 − DMEM + 10% FCS + 2 mM Gln: Schneider’s insect medium + 10 IU/mL penicillin + 20 μg/mL streptomycin). A total of 200 μL of culture medium was gently pipette-mixed with the pellet to create a suspension of cells, which were spread over poly-L-lysine-coated glass coverslips (5 × 20 mm) in 35 mm Petri dishes containing 2 mL locust culture medium. Petri dishes were incubated overnight at 36.5 °C in a 5% CO_2_ atmosphere.

### 4.3. Whole-Cell Patch-Clamp of Locust Neurons

Experiments were conducted 16–24 h after neuron dissociation. Coverslips covered in locust neurons were placed into a patch-clamp perfusion chamber, and were then constantly perfused with locust saline solution (180 mM NaCl, 10 mM KCl, 2 mM CaCl_2_, 10 mM HEPES, and pH 7.2 with NaOH). Borosilicate glass patch pipettes (1B150F-4, World Precision Instruments, Hertfordshire, UK), pulled to ~5–10 MΩ with a P-97 Flaming/Brown micropipette puller (Sutter Instrument Co., Novato, CA, USA), were filled with caesium pipette solution (140 mM CsCl, 1 mM CaCl_2_, 1 mM MgCl_2_, 11 mM EGTA, and 5 mM HEPES, pH 7.2) and placed in a patch pipette holder mounted on the patch-clamp amplifier head stage. Patch-clamped cells were clamped at a holding potential (V_H_) of −75 mV. Whole-cell currents were monitored by using an Axopatch 200A patch-clamp amplifier (Molecular Devices, San Jose, CA, USA) and recorded with WinWCP V4.5.7 software (Dr John Dempster, Institute of Pharmacy & Biomedical Sciences, University of Strathclyde, Glasgow, UK). Test compounds were focally applied to cells as 1 s pulses using a DAD-12 superfusion system (ALA Scientific Instruments, Farmingdale, NY, USA). Between each application of test compounds, clamped cells were washed for 1 min with locust saline. PhTX analogues and *d*-tubocurarine were co-applied with 100 μM ACh at concentrations in the range of 10 nM to 10 μM for all compounds, except for Cha-PhTX-12, which was applied at 10 pM to 10 μM.

### 4.4. Concentration–Inhibition Analysis

A total of 100 μM acetylcholine (ACh) was applied to cells twice prior to (control) and once after the co-application of 100 μM ACh and PhTX analogue. Peak current and current after 1 s of application were measured using WinWCP, and then the percentage of control response values were generated by normalising responses to ACh + PhTX co-application to the ACh-only control response. GraphPad Prism 9 (GraphPad Software, San Diego, CA, USA) was used for data analysis, graph plotting, curve fitting, and statistical analysis. To estimate the ACh EC_50_, concentration–response data were fit by the Hill equation:

(1) $$\%\;\mathrm{maximum}\;\mathrm{response}=\frac{100}{\left(1+10^{\left({\mathrm{LogEC}}_{50}-X\right)S}\right)}$$

where *X* is the log of the ACh concentration and *S* is the Hill slope. Concentration–inhibition curves were generated by plotting the mean and standard error of the normalised current measurements against log antagonist concentration, and then IC_50_ values were estimated by fitting a non-linear regression curve by using the equation:

(2) $$\%\;\mathrm{control}\;\mathrm{response}=\frac{100}{\left(1+10^{\left(X-{\mathrm{LogIC}}_{50}\right)S}\right)}$$

where *X* is the log of the concentration of the PhTX analogue and *S* is the Hill slope. The sum of squares F-test comparisons were run to evaluate differences in the IC_50_ values of each compound, or peak vs. 1 s current, with a *p*-value of <0.05 being considered as statistically significant.

## Figures and Tables

**Table 1:** IC_50_ values for inhibition of locust neuron nAChR by PhTX analogues.

Compound	IC_50_ (95% CI), μM		Peak/1 s Ratio
	Peak Current	1 s Current	
PhTX-343 (*n* = 15)	39.8 (12.0–132)	0.80 (0.48–1.34)	50 ^++++^
PhTX-12 (*n* = 19)	1.17 (0.72–1.92) ****	0.13 (0.08–0.21) ****	9.0 ^++++^
Cha-PhTX-343 (*n* = 10)	1.75 (0.83–3.68) ****	0.44 (0.22–0.89)	4.0 ^++^
Cha-PhTX-12 (*n* = 7)	0.0075 (0.0042–0.013) ****	0.0017 (0.0008–0.0035) ****	4.4 ^++^

**** *p* < 0.0001 vs. PhTX-343 IC_50_; ^++^ *p* < 0.01 & ^++++^ *p* < 0.0001 for peak vs. 1 s IC_50_.

## Data Availability

All data and supporting data are reported in this manuscript and supporting materials.

## Supplementary Materials

The following are available online, Scheme S1: Synthesis of compound Cha-PhTX-12. References [25,33] are cited in the supplementary materials.

## Sample Availability

Samples of the compounds are not available from the authors.
