# Supplementary material for: The Effects of Structural Alterations in the Polyamine and Amino Acid Moieties of Philanthotoxins on Nicotinic Acetylcholine Receptor Inhibition in the Locust, Schistocerca gregaria
Source: Molecules. 2021 Nov 19;26(22):7007. doi: 10.3390/molecules26227007 (PMC8622278; doi:10.3390/molecules26227007)

molecules
MDPI

*Supplementary Material for*

# The Effects of Structural Alterations in the Polyamine and Amino Acid Moieties of Philanthotoxins on Nicotinic Acetylcholine Receptor Inhibition in the Locust, *Schistocerca gregaria*.

**Victoria L. Luck[1], David P. Richards[1], Ashif Yasin Shaikh[2], Henrik Franzyk[2], and Ian R. Mellor[1*]**

[1] School of Life Sciences, University of Nottingham, University Park, Nottingham, NG7 2RD, UK.

[2] Department of Drug Design and Pharmacology, Faculty of Health and Medical Sciences, University of Copenhagen, Jagtvej 162, DK-2100 Copenhagen, Denmark; ashif@sund.ku.dk; henri.franzyk@sund.ku.dk

* Correspondence: ian.mellor@nottingham.ac.uk; +44 1159 513257

## Contents

Synthesis of Cha-PhTX-12........................................ 2

Analytical HPLC chromatogram........................................ 4

MALDI-TOF HR-MS spectrum........................................ 5

$^1$H NMR spectrum........................................ 6

$^{13}$C NMR spectrum........................................ 7

**List of abbreviations used:** Cha: cyclohexylalanine; DBU: 1,8-diazabicyclo[5.4.0]undec-7-ene; Pfp: pentafluorophenyl; r.t.: room temperature; TLC: thin-layer chromatography; VLC: vacuum liquid chromatography

molecules

**Scheme S1. Synthesis of compound Cha-PhTX-12**

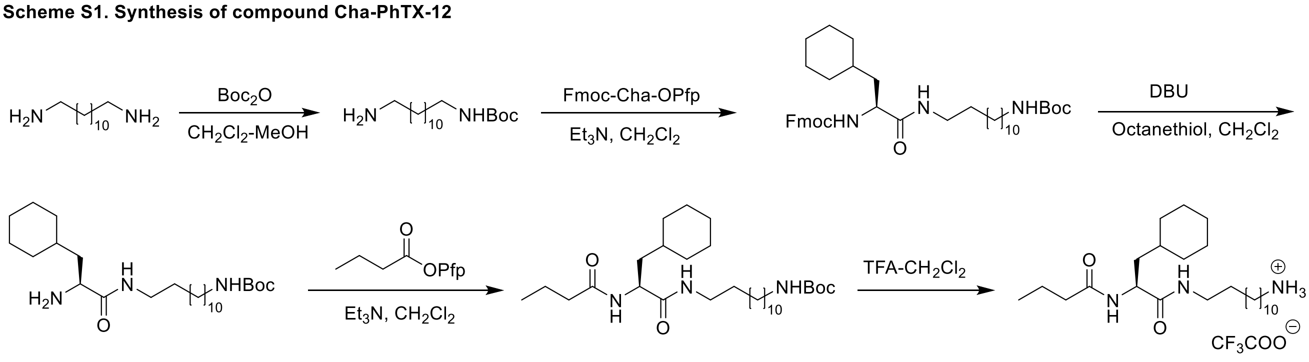


Cha = cyclohexylalanine
Pfp = pentafluorophenyl

## Synthesis of Cha-PhTX-12

Mono-Boc-1,12-diaminododecane [1] (1.00 g, 3.32 mmol) and Fmoc-Cha-OPfp [2] (2.04 g, 3.64 mmol) were successively dissolved in dry $CH_2Cl_2$ (20 mL), and then $Et_3N$ (0.46 mL, 3.32 mmol) was added. Upon stirring at r.t. for 3 h, TLC (hexane–EtOAc 2:1) showed a single spot at $R_f$ 0.4. The reaction mixture was diluted with EtOAc (100 mL), and then washed with $H_2O$ (2 × 50 mL), dried with $Na_2SO_4$, and the resulting filtrate was concentrated to give the crude product (1.88 g, 77%) as a colorless oily substance. The crude Fmoc-Cha-PhTX-12(Boc) (1.22 g, 1.66 mmol) was dissolved in dry THF (15 mL), and then octanethiol (16.6 mL, 10 × 1.66 mmol) and DBU (0.33 mL, 0.2 × 1.66 mmol) were added. After stirring for 1 h at r.t., TLC ($CH_2Cl_2$–MeOH–32% aq. $NH_3$ 200:10:1) showed full conversion into the product (seen at $R_f$ 0.71). The reaction mixture was concentrated in vacuo (to remove THF), and then the resulting residue was loaded onto a VLC column (5 × 5 cm; Merck 60H silica gel), which was eluted successively with petroleum ether (300 mL), petroleum ether–EtOAc (1:1, 400 mL; and 1:2, 300 mL), and $CH_2Cl_2$–MeOH–32% aq. $NH_3$ 200:10:1 (222 mL) to afford slightly impure $H_2N$-Cha-PhTX-12(Boc) (0.77 g, >99%) as a brownish foamy substance. Finally, $H_2N$-Cha-PhTX-12(Boc) (0.74 g, 1.63 mmol) was acylated for 20 h with $C_3H_7COOPfp$ [1] (0.48 g, 1.2 × 1.63 mmol) in dry $CH_2Cl_2$ (5 mL) with $Et_3N$ (0.23 mL, 1.63 mmol) added. When the reaction mixture was concentrated, TLC (petroleum ether– $Me_2CO$ 1:1) showed the product at $R_f$ 0.31. The residue was redissolved in $CH_2Cl_2$ (10 mL) and loaded onto a VLC column (4 × 4 cm; Merck 60H silica gel), which was eluted successively with hexane (150 mL) and hexane–EtOAc mixtures (10:1, 220 mL; 3:1 450 mL; 2:1, 150 mL; 1:1, 300 mL). The fractions containing the desired product were concentrated to afford slightly impure Cha-PhTX-12(Boc) (0.69 g, 81%) as yellowish foamy substance. Deprotection of Cha-PhTX-12(Boc) (0.69 g, 1.32 mmol) was performed in $CH_2Cl_2$–TFA (9:1, 5 mL) under stirring at r.t. for 3 h (RP-18 TLC with 60% MeCN–0.1% aq. TFA showed the product at $R_f$ 0.50). The reaction mixture was concentrated to dryness, and then the resulting residue was dissolved in 50% MeCN–0.1% aq. TFA (6 mL) and loaded onto a VLC column (7 × 4 cm; packed with Merck Lichroprep RP-18 40–63 µm), which was eluted with 0.1% aq. TFA (100 mL), 5% MeCN (in 0.1% aqueous TFA; 200 mL), 20% MeCN (in 0.1% aqueous TFA; 200 mL), 40% MeCN (in 0.1% aqueous TFA; 300 mL), 50% MeCN (in 0.1% aq. TFA; 300 mL) and 60% MeCN (in 0.1% aqueous TFA; 300 mL). Fractions containing the desired product were concentrated to afford slightly impure Cha-PhTX-12 (0.49 g; 70%). A pure

sample for testing was obtained by further purification via preparative reversed-phase high-pressure liquid chromatography (RP-HPLC) on a Phenomenex Luna C18(2) column (250 × 21.2 mm, particle size: 5 µm) on a Shimadzu system consisting of a CBM-20A Prominence communication bus module, two LC-20AP Prominence pumps, an SPD-M20A Prominence diode array detector, and an SIL- 20A HT Prominence autosampler. Elution was performed by using an aqueous MeCN gradient with 0.1% TFA (eluent A: 5:95 MeCN-$H_2O$:0.1% TFA, eluent B: 95:5 MeCN-$H_2O$:0.1% TFA (using a 0 → 100% B gradient during 20 min).

$^{1}$H NMR (600 MHz, methanol-$d_4$): δ 7.90 – 7.81 (m, 2H), 4.31 – 4.24 (m, 1H), 3.14 – 2.99 (m, 3H), 2.81 (t, $J$ = 7.7 Hz, 2H), 2.11 (td, $J$ = 7.2, 1.0 Hz, 2H), 1.70 – 1.64 (m, 2H), 1.64 – 1.59 (m, 3H), 1.59 – 1.49 (m, 5H), 1.49 – 1.44 (m, 1H), 1.44 – 1.40 (m, 1H), 1.40 (d, $J$ = 5.8 Hz, 1H), 1.37 (d, $J$ = 6.5 Hz, 1H), 1.32 – 1.26 (m, 4H), 1.24 (d, $J$ = 8.4 Hz, 5H), 1.22 – 1.20 (m, 7H), 1.20 – 1.06 (m, 4H), 0.93 – 0.77 (m, 6H).
$^{13}$C NMR (151 MHz, methanol-$d_4$) δ 174.6, 173.5, 51.1, 48.2, 39.4, 39.3, 38.9, 37.3, 34.1, 33.4, 32.1, 29.3, 29.2, 29.2, 29.1, 29.0, 28.9, 28.8, 27.2, 26.5, 26.2, 26.0, 25.9, 18.9, 12.6.
HRMS for $C_{26}H_{51}N_3O_2$ $[M+H]^+$ Calcd: 424.3903, found: 424.3910

**References:**

[1] Wellendorph, P.; Hansen, S. H.; Jaroszewski, J. W.; Franzyk, H. A Sequential High-Yielding Large-Scale Solution-Method for Synthesis of Philanthotoxin Analogues. *Eur. J. Med. Chem.* **2003**, *38*, 117-122.

[2] Olsen, C. A.; Mellor, I. R.; Wellendorph, P.; Usherwood, P. N. R.; Witt, M.; Franzyk, H.; Jaroszewski, J. W. Tuning Wasp Toxin Structure for Nicotinic Receptor Antagonism: Cyclohexylalanine-Containing Analogues as Potent and Voltage-Dependent Blockers. *ChemMedChem* **2006**, *1*, 303-305.

**Analytical HPLC chromatogram:**

Analytical HPLC was performed on a Phenomenex Luna Omega Polar C18 column (150 × 4.6 mm; particle size: 3 µm; pore size: 100 Å) using a Shimadzu Prominence and Shimadzu Nexera system. Gradient elution: 0 → 100% B during 15 min

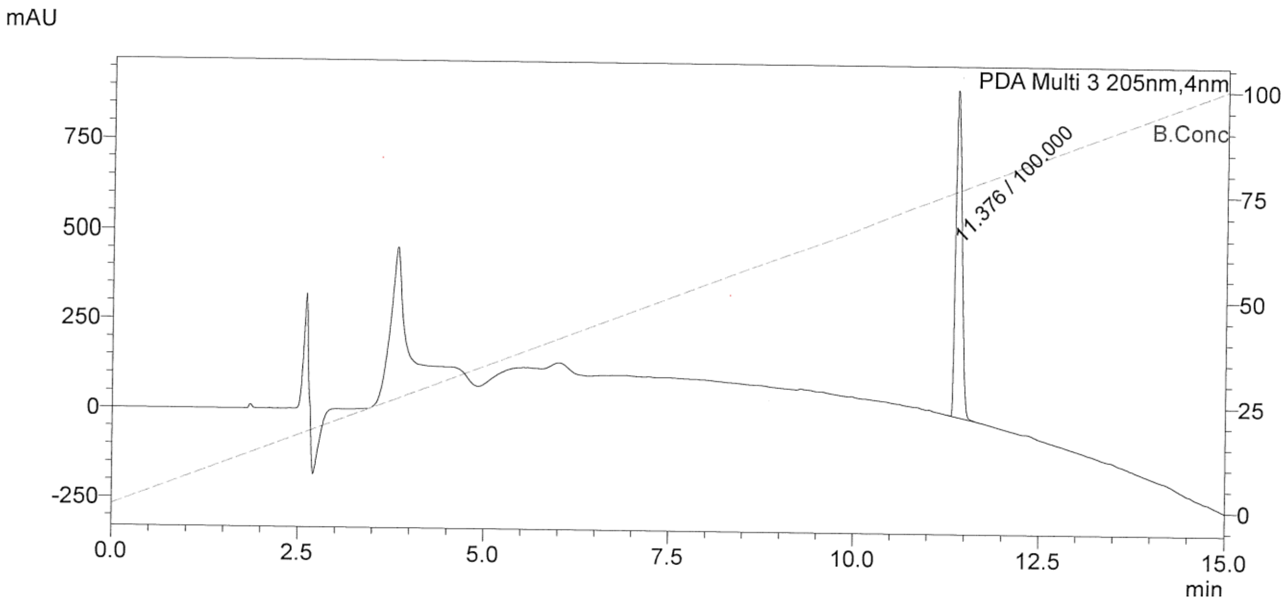

molecules

MDPI

**MALDI-TOF MS spectrum:**

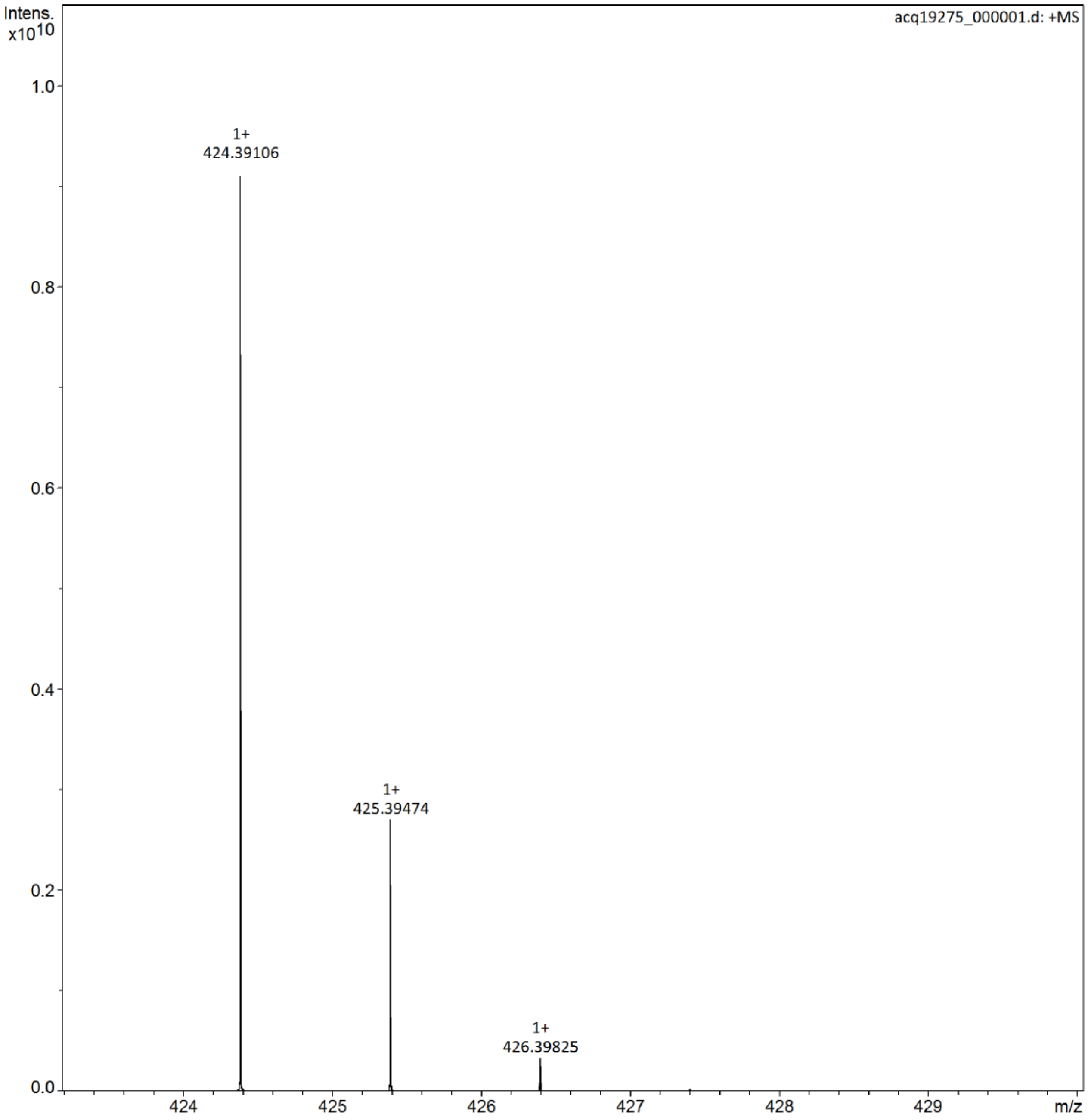

## $^{1}$H NMR spectrum:

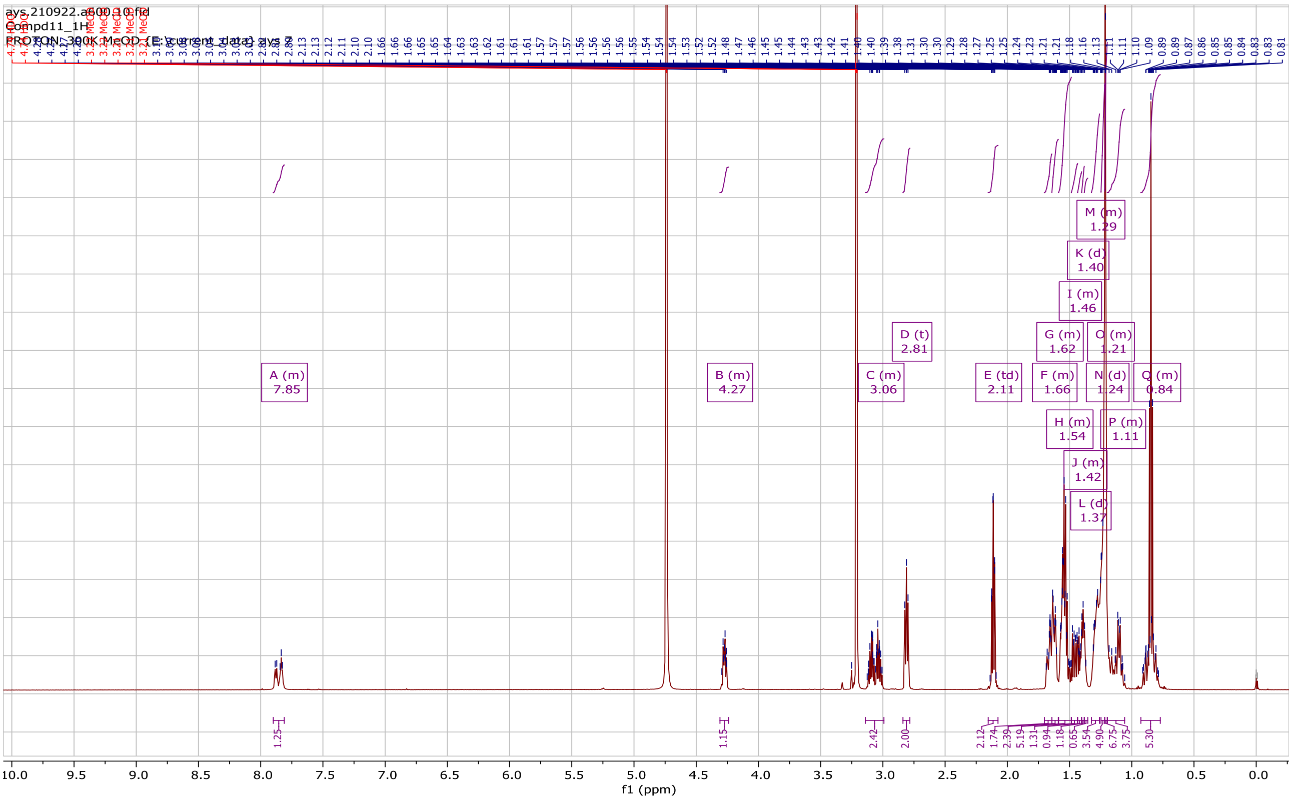

molecules

MDPI

**$^{13}$C NMR spectrum:**

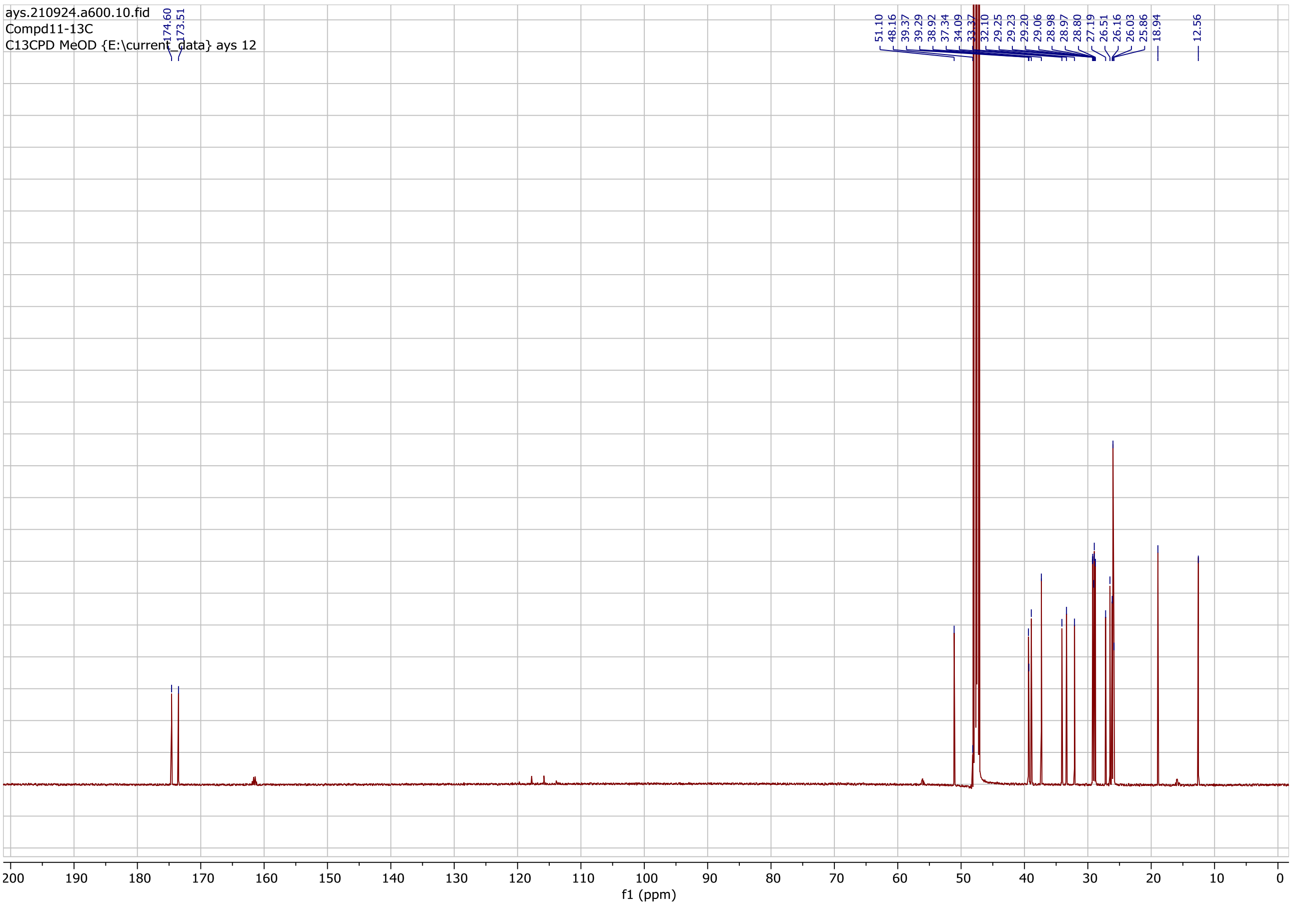

Supplement: Supplementary file 1 [file molecules-26-07007-s001.zip › molecules-1450176-rsb-sp.pdf]
